# A Rare Case of Parvovirus B19 Infection Induced Paroxysmal Cold Hemoglobinuria in an Adult Female

**DOI:** 10.7759/cureus.11622

**Published:** 2020-11-22

**Authors:** Neenu Kuruvilla, Vishnu Vinay, Rahul Rajendran, Irshad Ali KM, Sheela Kurian

**Affiliations:** 1 Internal Medicine, Government Medical College Kottayam, Kottayam, IND

**Keywords:** paroxysmal cold hemoglobinuria (pch), parvovirus b19, autoimmune hemolytic anemia (aiha), rare

## Abstract

Paroxysmal cold hemoglobinuria (PCH) is a rare form of autoimmune hemolytic anemia (AIHA). PCH occurs in acute and chronic forms. The main risk factors for PCH include viral infections, vaccination, and syphilis. PCH presentations are common in the pediatric population. The occurrence of PCH following parvovirus B19 infection in adults is rare. We report a case of a 23-year-old female who presented with giddiness, fatigue, greying of vision, and presyncope for four days, on subsequent evaluation was found to have evidence of hemolysis and bone marrow suppression. Parvoviral intranuclear inclusions were detected in bone marrow biopsy and parvoviral B19 IgM antibody was detected. Donath Landsteiner antibody test was also positive. Hence a diagnosis of PCH secondary to parvovirus B19 infection was made. She was started on pulse dose steroids and intravenous immunoglobulin (IVIG) and showed significant improvement.

## Introduction

Paroxysmal cold hemoglobinuria (PCH) is a rare form of autoimmune hemolytic anemia (AIHA), characterized by biphasic, polyclonal IgG autoantibody that binds specifically to the P antigen of RBCs [1]. This binding occurs at a lower temperature leading to complement system activation and red cell lysis at 37°C. The IgG autoantibody involved is Donath- Landsteiner (DL) antibody [2]. PCH is more common in the pediatric population [3]. PCH can occur in both acute and chronic forms. The important risk factors for acute PCH include viral infections (mumps, measles, chickenpox, Epstein-Barr virus, cytomegalovirus, influenza, parvovirus B19, coxsackievirus A9, and adenovirus) and vaccination (measles) [4,5]. The occurrence of parvovirus B19 infection predisposing to acute PCH in adults is rare. Here we report such a rare case of parvovirus B19 induced acute PCH.

## Case presentation

A 23-year-old Indian female, with no significant past medical history and family history, presented to our hospital with giddiness, fatigue, greying of vision, and presyncope for four days. There was no history of fever, chest pain, palpitation, shortness of breath, pedal edema, abdominal pain, vomiting, hematemesis, haematuria or, malena. She denied any history of alcohol intake or substance abuse. Her menstrual cycles were regular with no history of menorrhagia or polymenorrhoea. The patient was on a non-vegetarian diet. On examination, she was conscious and oriented, with a temperature of 37°C, pulse rate of 114/min, blood pressure of 110/60 mm Hg, respiratory rate of 18/min, and SpO2 of 96% in room air. Physical examination showed the presence of pallor in the conjunctiva, nailbed, and palms. Systemic examination was unremarkable except for a systolic flow murmur. Labs at presentation were significant for bicytopenia (Table 1).

**Table 1:** Labs at presentation MCV: mean corpuscular volume; ESR: erythrocyte sedimentation rate;  MCHC: mean cell hemoglobin concentration;  MCH: mean cell hemoglobin; ALT: alanine transaminase; AST: aspartate transaminase; ALP: alkaline phosphatase

Variable	Measurement	Reference Values
Hemoglobin (g/dL)	6.7	12-16
Total leucocyte count (/mm^3 ^)	2400	4000-11,000
Neutrophils (%)	36	50-70
Lymphocytes (%)	46	30-45
Platelet count (/mm^3^)	2,05,000	1,50,000-4,50,000
ESR (mm/h)	148	0-20
MCV (microm^3^)	99	80-98
MCHC (g/dL)	34	33-36
MCH (pg/cell)	34	28-32
Urea (mg/dL)	16	8-20
Creatinine (mg/dL)	0.5	0.5-1.1
Total bilirubin (mg/dL)	1.3	0.3-1.0
Direct bilirubin (mg/dL)	0.3	0.1-0.3
ALT (units/L)	36	10-40
AST (units/L)	28	10-40
ALP (units/L)	66	30-120

ECG and chest X-ray were normal. ultrasound of abdomen showed no hepatosplenomegaly. On the second day of hospital stay, she collapsed suddenly. On examination, she was tachycardic and hypotensive with a pulse rate of 112/min, and blood pressure of 80/60 mm Hg. Labs showed a rapid decline in hemoglobin and evidence of hemolysis (Table 2). She was transferred to the ICU, transfused with two units of packed red cells, and was started on pulse dosage steroids with intravenous methylprednisolone 1000 mg once daily. There was no history of cold shower or swimming that led to a rapid change in body temperature.

**Table 2:** Labs on Day 2 showed rapid decline in hemoglobin level, evidence of hemolysis and low reticulocyte count TB: total bilirubin; DB: direct bilirubin; DCT: direct Coombs test; LDH: lactate dehydrogenase; TSH: thyroid stimulating hormone; TIBC: total iron binding capacity; ANA-IF: antinuclear antibody immunofluorescence

Variable	Measurement	Reference values
Hemoglobin (g/dl)	2.7	12-16
Total leucocyte count	3700	4000-11,000
Neutrophils (%)	63	50-70
Lymphocyte (%)	28	30-45
Platelet count	2,24,000	1,50,000-4,50,000
Total bilirubin (mg/dL)	2.4	0.3-1.0
Direct bilirubin (mg/dL)	0.4	0.1-0.3
Reticulocyte count (%)	0.1	0.5-1.5
LDH (units/L)	696	80-225
TSH (micro Units/mL)	0.9	0.5-4.0
Serum vitamin B12 (pg/mL)	400	200-800
Serum folate (ng/mL)	12	1.8-9.0
Serum iron (microg/dL)	80	50-150
TIBC (microg/dL)	300	250-310
Transferrin saturation (%)	30	20-50
Serum ferritin (ng/mL)	100	11-307
ANA-IF	Negative	

**Figure 1:**
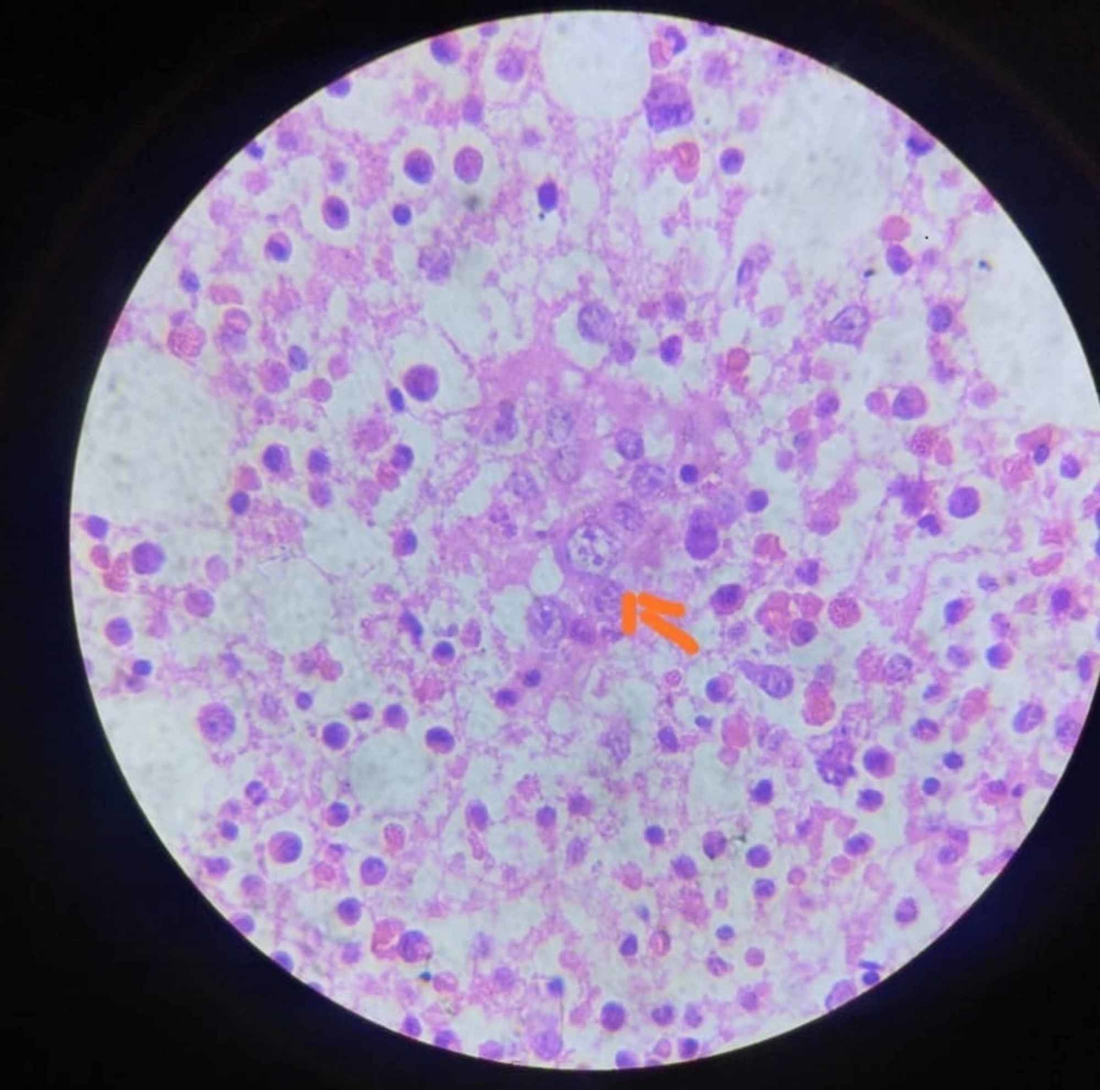
Bone marrow biopsy showing parvoviral intranuclear inclusions Arrow-head shows parvoviral intranuclear inclusion inside the proerythroblast cell.

Peripheral smear showed normocytic normochromic anemia and leucopenia with a neutrophilic predominance. In view of severe bicytopenia and low reticulocyte count, bone marrow biopsy was done and revealed evidence of proerythroblasts with parvoviral intranuclear inclusions (Figure 1). Parvovirus B19 IgM was found to be positive. Hence she was initiated on intravenous immunoglobulin (IVIG) and continued for five days.

**Figure 2:**
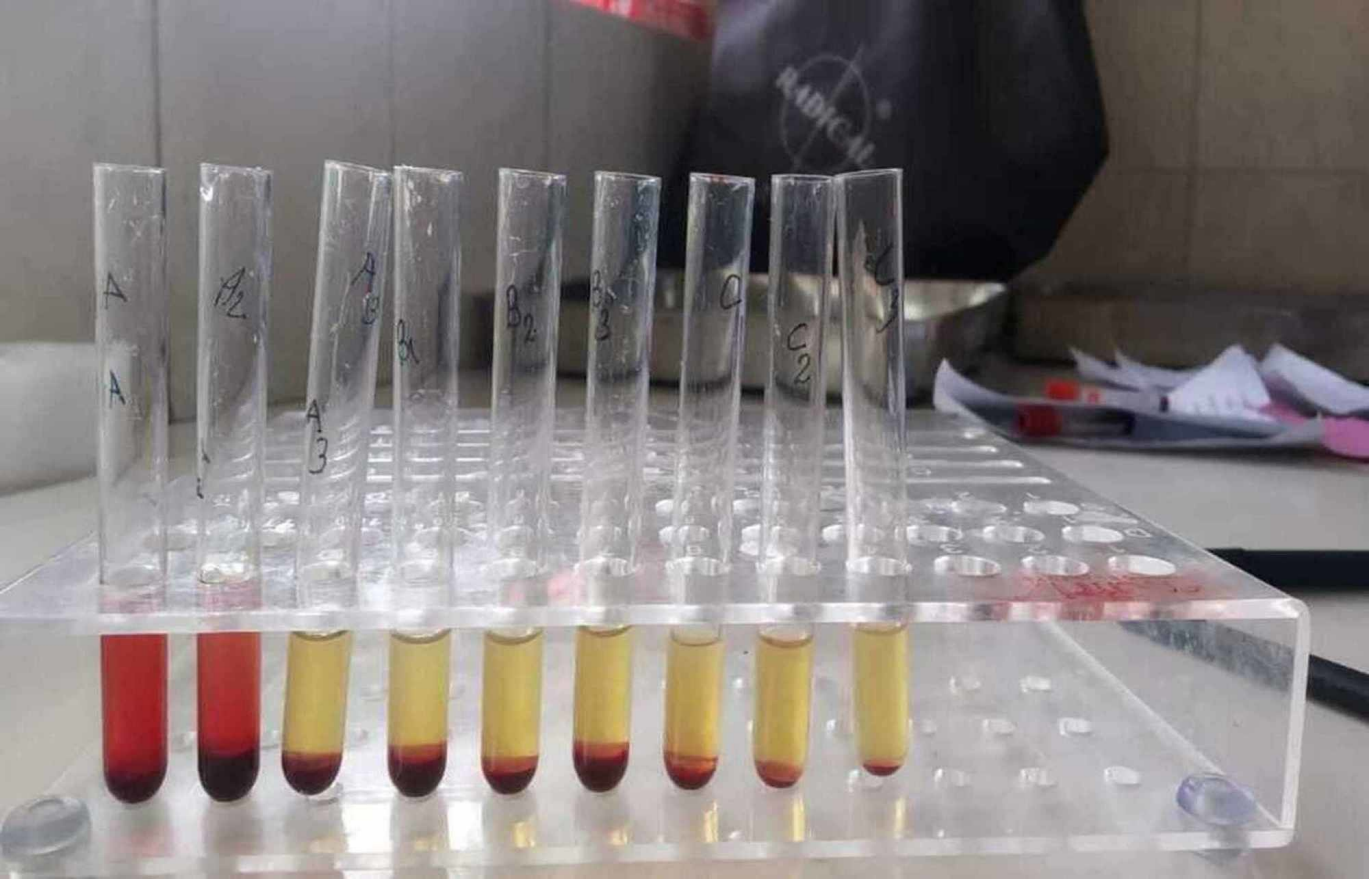
Donath Landsteiner test

**Figure 3:**
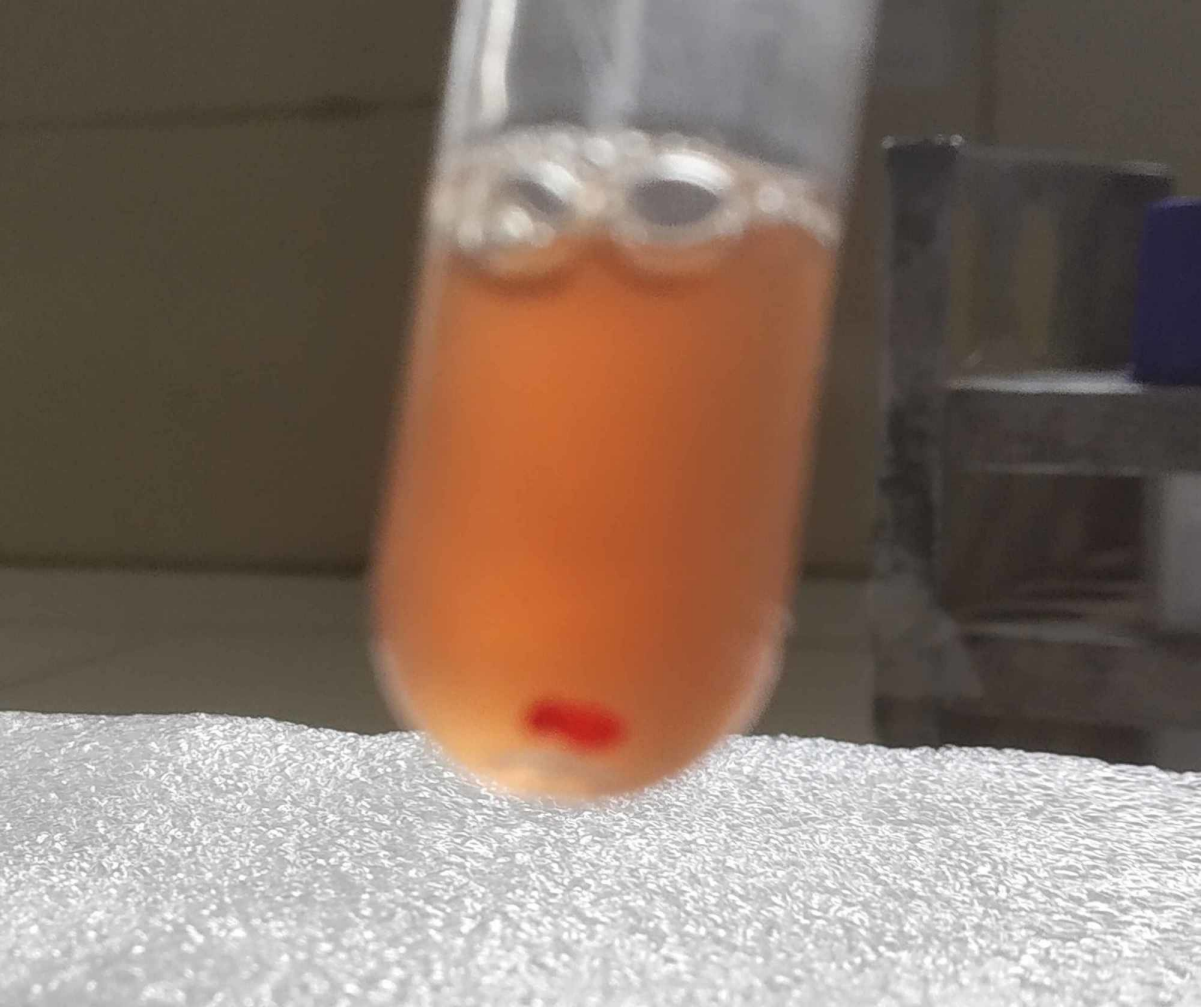
Positive Donath Landsteiner test

A hemolytic anemia workup was done to confirm the type of autoimmune hemolytic anemia. Direct Coombs test (DCT) for monospecific and polyspecific IgG/C3d was positive. The thermal amplitude of the antibody was found to be 4+ at 4°C, 2+ at 22°C, and reactive at 37°C, with evidence of hemolysis. Cold agglutination titer performed in normal saline plain gel card was found to be negative. DL test was done (Figure 2) and showed a positive result (Figure 3). Hence a diagnosis of PCH secondary to parvoviral B19 was made. 

She was continued on IVIG and steroids. Her general condition improved over the next one week. The total duration of hospital stay was four weeks. She was discharged on a tapering dose of steroids with oral prednisolone 50 mg once daily.

## Discussion

PCH is an AIHA characterized by DL antibody causing red cell lysis [6]. The occurrence of PCH is rare, accounting for less than 1% of all autoimmune hemolytic anemias [7]. PCH causes complement fixation at low temperatures, subsequently leading to intravascular hemolysis on rewarming. PCH can present in both acute and chronic forms. When PCH was first identified, it was described as a chronic condition in adults with tertiary syphilis [5]. With the advent of effective treatment for syphilis, the chronic relapsing form of PCH became rare [8]. Nowadays, the common presentation of PCH is an acute transient non-recurring illness.

Acute PCH predominantly occurs in the pediatric population with a recent history of viral illnesses or following immunization [9]. The incidence in pediatric groups is 0.001/100000 per year in boys and 0.0005/100000 per year in girls [10,11]. The mean age of onset is 3.8 years [8]. Certain viruses have been implicated in precipitating episodes of PCH in children, including measles, mumps, varicella, cytomegalovirus, Epstein - Barr virus, influenza virus, parvovirus B19, coxsackie, and adenovirus [12]. The mechanism of how an infectious agent induces PCH is poorly understood. One proposed theory suggests that viruses alter the glycoproteins on the erythrocyte membrane which further stimulates autoantibody formation [4]. Another theory points that molecular mimicry between self-antigens and foreign antigens leads to the production of cross-reactive antibodies [4].

Typical clinical features include fever, chills, abdominal pain, and hemoglobinuria on exposure to cold. Hemoglobinuria can persist for several months despite the resolution of intravascular hemolysis. Physical examination may show fever, pallor, icterus, and abdominal tenderness [13]. Laboratory findings include features of red blood cell lysis like indirect hyperbilirubinemia, low haptoglobin, decreased complement, elevated LDH, reticulocytosis, and hemoglobinuria. Hemosiderinuria is also characteristic but usually develops three to four days after the onset of hemolysis. The pathognomonic finding is erythrophagocytosis by neutrophils in peripheral blood smear [5]. The diagnosis of PCH is confirmed by the presence of DL antibodies [14]. The DL antibody fixes the first two components of the complement cascade and dissociates upon rewarming. This results in complement-mediated intravascular hemolysis. The original assay for the DL test used patient serum along with test RBCs and a pooled human serum. These are incubated at 4°C to allow the antibody to bind and subsequently fix the complements. This is then transferred to 37°C to allow for the latter components of complement to be activated and leading to hemolysis. As a control, hemolysis does not occur if the reaction mixture is maintained continuously at 37°C because the DL antibodies require cold temperature for RBC sensitization. To demonstrate the P antigen specificity of DL antibody, ABO-compatible, P antigen-negative RBCs may be used as a negative control.

 Because of the biphasic nature, the direct antiglobulin test (DAT) is negative for IgG and positive for anti-C3 [4]. Cold agglutinin disease is one of the main differential diagnoses. But it can be distinguished from PCH by a negative DL test, peripheral blood smear showing RBC agglutination, and a positive serum IgM. 

The treatment of an acute episode of PCH is generally supportive, which includes analgesics and avoidance of cold exposure. Individuals with severe hemolysis may require adequate hydration, blood transfusions, and glucocorticoids [15]. Blood transfusions must be administered using a blood warmer to prevent the binding of autoantibody to the transfused cells [16]. For patients with on-going hemolysis, more potent immunosuppressive therapies, and anti-complement therapies like treatment with rituximab, cyclophosphamide or azathioprine may be helpful [17]. Plasmapheresis can also be used to lower the antibody titer [18].

## Conclusions

PCH in adults, with parvovirus B19 precipitating the same, is rare. It can occur in acute and chronic forms. Acute forms are more common in the pediatric population, with viral infections and vaccinations being the major etiologies. Chronic forms are rare in the modern era due to effective treatment for syphilis. The diagnosis of PCH is confirmed by the presence of DL antibodies. PCH is treated supportively, with analgesics and avoidance of cold exposure.
